# Gradual vs. Maximal Acceleration: Their Influence on the Prescription of Maximal Speed Sprinting in Team Sport Athletes

**DOI:** 10.3390/sports6030066

**Published:** 2018-07-21

**Authors:** Warren B. Young, Grant M. Duthie, Lachlan P. James, Scott W. Talpey, Dean T. Benton, Anthony Kilfoyle

**Affiliations:** 1 School of Health and Life Sciences, Federation University, Ballarat 3350, Australia; l.james@latrobe.edu.au (L.P.J.); talpeys1@southernct.edu (S.W.T.); tonykilfoyle@hotmail.com (A.K.); 2 School of Exercise Science, Australian Catholic University, North Sydney 2060, Australia; grant.duthie@acu.edu.au; 3 Department of Rehabilitation, Nutrition and Sport, School of Allied Health, La Trobe University, Melbourne 3086, Australia; 4 Exercise Science Department, Southern Connecticut State University, New Haven, CT 06515, USA; 5 Atletico, Melbourne, Australia; dbenton@atletico.com.au

**Keywords:** acceleration, team sport, conditioning, programming

## Abstract

The primary purpose of this study was to determine if a difference existed between peak speed attained when performing a sprint with maximal acceleration versus from a gradual build-up. Additionally, this investigation sought to compare the actual peak speed achieved when instructed to reach 75% and 90% of maximum speed. Field sport athletes (*n* = 21) performed sprints over 60 m under the experimental conditions, and the peak speed was assessed with a radar gun. The gradual build-up to maximum speed (8.30 ± 0.40 m∙s^−1^) produced the greater peak speed (effect size = 0.3, small) than the maximum acceleration run (8.18 ± 0.40 m∙s^−1^), and the majority of participants (62%) followed this pattern. For the sub-maximum runs, the actual mean percentage of maximum speed reached was 78 ± 6% for the 75% prescribed run and 89 ± 5% for the 90% prescription. The errors in attaining the prescribed peak speeds were large (~15%) for certain individuals, especially for the 75% trial. Sprint training for maximum speed should be performed with a gradual build-up of speed rather than a maximum acceleration. For sub-maximum interval training, the ability to attain the prescribed target peak speed can be challenging for field sport athletes, and therefore where possible, feedback on peak speeds reached should be provided after each repetition.

## 1. Introduction

The ability to accelerate in relatively short sprints and to repeat sprint efforts are considered important in field sports such as various football codes [1]. However, the need to train and assess maximum speed is not so clear because it is argued that sprints are rarely long enough in competition to reach top speed [2,3]. Despite there being no consensus of what constitutes a sprint or acceleration effort in team sports quantified using tracking devices, previous research has identified mean sprint durations of ~3 s [4]. While the aforementioned argument may hold true for sprints performed from a stationary start, in field sports it is more common for sprints to begin from a moving start such as a walk or run [5,6,7,8]. In one study investigating the effect of pre-sprint speed on the distance required to reach maximum or near maximum speed [9], twelve semi-elite Australian football players were required to sprint maximally for 60 m while running speed was recorded continuously. The distance required to reach various percentages of maximum speed was measured in sprints performed from a standing start, and following an approach of 3 m·s^−1^ (“jogging”), 5 m·s^−1^ (“striding”), and 7 m·s^−1^ (“fast striding”). On average, it took 26.6 m, 23.4 m, 21.9 m, and 15.8 m from the beginning of the sprint to reach 97.5% of maximum speed, respectively. These results indicated that the faster the locomotion of the athlete prior to a maximum effort sprint, the shorter the distance required to reach near maximum speed. Further, this investigation revealed that only 20 m was required to reach 98.5% of maximum after a fast striding approach run. The same method was used with professional rugby union players [5], and it was reported that a jogging or striding start resulted in substantially less time to reach maximum speed than from a standing start. Subsequently, an analysis of competitive professional rugby union games was performed on 28 players, and revealed that more than 50% of the sprints were commenced from a moving start (walking to striding). The authors concluded that sprints in competition frequently permitted the attainment of 90–99% of maximum speed for both forwards and backs, and suggested that players should regularly perform efforts in training that allow near maximum speed to be achieved [5].

Time–motion analysis of field sports, such as rugby league, rugby union, soccer, Gaelic football and American football, indicates that sprinting only accounts for about 2–6% of total game time [10,11,12,13,14,15,16]. However, this does not diminish the potential that sprinting speed has on match performance. It is possible that maximum speed in sprints has a disproportionately higher impact on performance. For example, in rugby union or rugby league, if an attacking player breaks through a defensive line, there may be an open field in which to sprint to score a try. In this situation, the maximum speed of the player in possession of the ball, as well as the speed of an opponent who is chasing, will likely be a key determinant of the scoring outcome. Unfortunately, research has neglected to address this issue, so no evidence is available to substantiate this notion. Nevertheless, prescription of sprint training that targets both acceleration and maximum speed for field sports seems justified.

One way to train for maximum speed development is to accelerate maximally from a stationary start, ensuring the distance of the sprint is long enough to reach an athlete’s maximum. An alternative approach is to gradually build up running speed with a sub-maximum acceleration [5] and then hold the peak for 10–20 m. Although it has been suggested the sub-maximum acceleration may prevent an “excessive anaerobic cost” [17], an analysis of these two approaches has not been conducted. It would be useful for coaches to know if the approach used to develop sprint speed has an effect on the peak speed that can be attained. If a higher peak speed can be reached in sprint training, an improved training stimulus may be expected.

Given that much of the locomotion in field sports is performed below maximum intensity, it is common for coaches to also prescribe “tempo” interval runs comprising sub-maximum efforts over various distances from 60–300 m to develop cardio-metabolic parameters [17]. For example, a percentage of maximum speed is typically used to prescribe the target speed for each effort, e.g., 75% or 90% of maximum. Since the prescribed speed represents the intensity of the effort and intensity is a fundamental training variable, it is of practical interest for coaches to know whether athletes can easily achieve a running speed prescribed to them. If there is a meaningful gap between a prescribed and actual running speed performed, the targeted training stimulus may not be achieved. Therefore, the objectives of this study were twofold. First, to determine if there is a difference in maximum speed attained when commencing a sprint from a maximum acceleration or from a more gradual build-up. It was hypothesized that a gradual build of speed would yield greater peak speeds. Second, to determine the error in attaining the prescribed peak speeds for efforts of 75% and 90% of maximum. The findings of this study will provide information to inform strength and conditioning coaches about how sprint training should be prescribed and coached to field-sport athletes. 

## 2. Materials and Methods

### 2.1. Experimental Approach to the Problem

This cross-sectional investigation sought to compare radar acquired peak speeds during 60-m sprints between gradual build-up and maximum acceleration conditions. The gradual build-up sprint was performed first, and a 5-min recovery was allowed to prevent fatigue from influencing the next sprint. Additionally, potential differences were assessed between prescribed and actual peak running speeds when subjects performed a 60-m sprint from a gradual acceleration aiming to reach 75% and 90% of their individual maximum speed in non-randomized order.

### 2.2. Subjects

Twenty-one males aged between 18 and 25 who participated in regional-level field sports mainly comprising soccer, hockey and Australian football were recruited to participate. As such, participants were experienced with both maximal and prescribed intensity running. They were required to be free of injury and illness at the time of the study so they were able to sprint with no restrictions. All subjects provided informed consent, and the study was approved by the University Human Ethics Committee. 

### 2.3. Procedures

Sprint testing was conducted on a natural grass field, with subjects wearing shoes with spikes or studs to prevent slippage. A standardized warm-up was used consisting of jogging, dynamic stretches of the lower extremities, and three single effort 60-m runs at increasing speeds. Subjects were instructed to run at 60%, 75%, and 90% of maximum speed, all with walk-back recoveries. The 75% and 90% runs were recorded for the purposes of comparing prescribed and actual peak running speeds. All sprints were commenced from a standing start, and subjects were allowed to commence each run when they were ready; that is, without the need to react to a starting signal. For the gradual build-up condition, subjects were instructed to gradually build up speed to reach a maximum before the 60-m mark. Although non-standardized instructions were given for the gradual buildup, all participants were familiarized throughout routine use of this approach during training. For the maximum acceleration sprint, the instruction was to accelerate as hard as possible from the start and sprint to the 60-m mark.

A calibrated radar gun (Stalker Professional Sports, Richardson, TX, USA, range 8.05–402.34 km/h, accuracy ± 0.1 km/h) sampling at 31.25 Hz was used to assess all peak speeds. Peak speeds were analyzed with Stalker ATS software (version 5.0, Stalker, Richardson, TX, USA) and a “heavy” filter was used to smooth the data and minimize signal noise. The radar gun was mounted on a tripod 2-m behind the start line and 1.2-m above the ground, and pointed at the back of the subject who ran in a straight line down a 1.2-m wide lane. Such instrumentation has displayed perfect validity when compared to photocell timing, alongside a test-retest reliability that achieved an intraclass correlation coefficient of 0.96–0.99, and a coefficient of variation of 0.7–1.9% [18].

### 2.4. Statistical Analyses

To decrease non-uniformity, raw data were log transformed prior to statistical analysis. Standardized differences were established from the log transformed data. Where applicable the data were back transformed to present mean and standard deviation values. Linear mixed models (lmer package in R; V 1.0.136.) were used to determine the magnitude of difference between the maximum speed achieved during a gradual or maximum acceleration effort. They were also used to determine the magnitude of difference between the prescribed running speed and the actual running speed achieved. The random effect in the models’ design was the athlete identification. The least squares mean test provided pairwise comparisons that were further assessed using magnitude based inferences [19]. Differences were described using standardized effect sizes (ES) and 90% confidence intervals (CI), categorized using the thresholds of: <0.2 trivial, 0.21–0.60 small, 0.61–1.20 moderate, 1.21–2.0 large and >2.0 very large [19]. Differences were considered real if there was a >75% likelihood of the observed effect exceeding the smallest worthwhile difference (0.20× between subject SD for each given speed), and are described as : 75–95%, likely, 95–99% very likely and >99%, almost certainly [20].

## 3. Results

**Figure 1:**
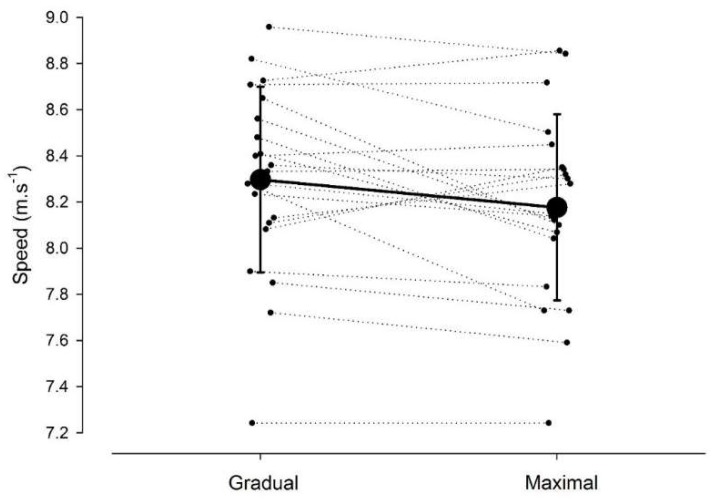
The maximal speed (m∙s^−1^) achieved when accelerating using a gradual versus maximum build-up. Individual athlete speeds are represented along with the mean and SD (error bars) for each conditions.

Raw data for the maximum speeds achieved during the gradual and maximal acceleration are provided in Figure 1. Overall, the mean ± SD maximum speed achieved was 8.34 ± 0.40 m∙s^−1^. There was *likely* a small difference (ES, 0.3; 90% CI, 0.1–0.5) between the maximum speed achieved when using a gradual (8.30 ± 0.40 m∙s^−1^) versus maximum (8.18 ± 0.40 m∙s^−1^) build-up, with a 75% likelihood that the gradual build-up resulted in a substantially greater maximum speed.

**Figure 2:**
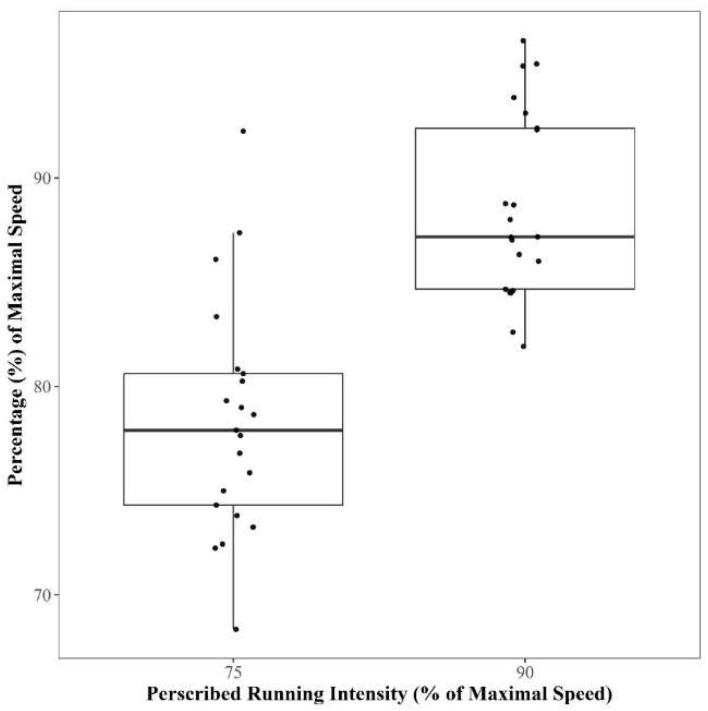
Actual speed achieved during sub-maximum running efforts (75% and 90% of maximum speed). Individual athlete speeds are represented and the box plot represents the median, interquartile ranges and 90% confidence limits of the group values.

**Figure 3:**
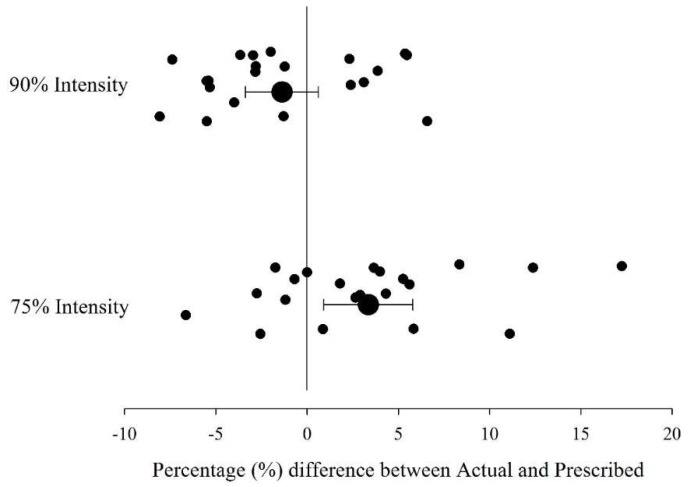
Individual and group (ES ± 90% CL) percentage differences in prescribed versus actual running intensities.

Figure 2 shows the actual running speeds expressed as a percentage of maximum speed. During the 75% of maximum speed effort, the athletes achieved a mean speed of 78 ± 6%, while for the 90% effort they achieved 89 ± 5%. The minimum and maximum individual values were 68% and 92% of maximum, respectively, for the 75% run; and 82% and 97% of maximum, respectively, for the 90% run. Figure 3 shows the difference between the prescribed and actual speeds achieved during submaximal runs. There was *very likely* a moderate difference (0.9; 0.3–1.3) between the prescribed (6.25 ± 0.30 m∙s^−1^) and actual (6.52 ± 0.38 m∙s^−1^) running speeds when running at 75% of maximal speed. There was *likely* a small difference (0.5; 0.0–0.9) between the prescribed (7.50 ± 0.36 m∙s^−1^) and actual (7.38 ± 0.36 m∙s^−1^) running speeds when running at 90% of maximal speed.

## 4. Discussion

The primary objectives of this study were to determine: (i) whether higher sprinting speeds are reached during a gradual buildup versus a maximal acceleration start; and (ii) if relevant differences existed between prescribed and actual speeds during submaximal running. The present findings demonstrate that greater maximal sprinting speeds could generally be achieved when a moving approach was used. Furthermore, likely differences were present between prescribed and actual running intensities. This information can be considered by coaches when programing training for field sport athletes.

This is the first study to determine if the approach to reaching maximum sprinting speed influences the peak speed achieved. The gradual build-up condition produced a 0.12 m·s^−1^ higher peak speed compared to the maximum acceleration condition. Further, 13 out of the 21 subjects (62%) achieved greater maximum speeds with the gradual build-up. These data suggest that, if targeting maximal speed development (instead of acceleration ability), then a gradual build up provides a desirable stimulus, as it allows individuals to obtain higher sprint speed. This approach also holds an increased specificity to the sprinting demands of many field sports, where sprints are rarely initiated from a standing start. For example, elite-level rugby league players are more than twice as likely to initiate a sprint while already moving forward (57.7% of occasions) when compared to stationary standing (24.3% of occasions) [6]. However, it is important for coaches to recognize the unique positional demands within their sport as this may affect the proportion of time sprints are initiated from a moving approach [5,6].

While the mechanism for the difference in peak speed was not examined and remains elusive, insights may be found by exploring the effect of instructions on force production. During maximum isometric contractions of the plantar flexors, an instruction to contract as hard as possible without concern for speed, produced much higher peak force (~70%) than an instruction to contract as fast as possible [21]. It may be such that, when compared to an action performed with maximal intent, a slower ramped contraction enables individual motor units to attain a higher peak force before additional motor units are recruited (as the recruitment threshold is lowered for explosive efforts) [22]. These factors would lead to a summation of motor units that result in greater peak force (albeit reduced rate of force development) during actions with a ramped rise in effort, and may therefore explain the faster sprint times when a gradual build-up is performed. However, it is important to consider that contribution of the mechanism to the observed performance differences is speculative and requires further exploration. 

Often, in training, team sport athletes are given a prescribed speed based on the subjective effort rating relative to a maximal effort [23]. This is indicative of team sports given that hand held timing is difficult when working with multiple athletes. The results of this study show that although average speeds were close to the prescribed, there were large individual differences observed which might make this approach difficult to implement accurately during training sessions. For example, some subjects underestimated the prescribed speed by 7%, while other overestimated by a similar amount for the 90% prescribed speed. This variation was even greater for the 75% trial, with one individual reaching as high as 92% of maximum, and another reaching only 68% of maximum. These results indicate that it can be challenging for field-sport athletes to achieve the prescribed peak speed, especially for a lower speed of 75% maximum. Developments in player tracking devices with real time feedback [24] may assist in ensuring athletes are achieving prescribed running intensities. Finally, it should be noted that as peak speeds were used in this present study, the average speed over a distance of interest was not considered. It is possible that average speeds may yield contrasting results.

An inability to regularly achieve a prescribed peak speed in training could be expected to produce a different training stimulus to what is intended. For example, if tempo runs at 75% were prescribed, but an athlete consistently reached speeds near 90% of maximum, premature fatigue would be expected to compromise the objective of the session. Additionally, such a disconnect between the prescribed load and the internal stress experienced by the athlete can compound over time, potentially resulting in a maladaptive response [25]. Since running-based conditioning through game-play is popular with coaches of field sports, the athletes in the present study may have had limited experience with interval training. Nevertheless, the results highlight the need for the coach to reinforce the importance of attaining the prescribed speed during interval training. As GPS is now commonplace at the professional level, these devices could be used to provide real time feedback on the speeds attained by team sport athletes to ensure they are achieving the desired speed.

From a practical perspective, to optimize the maximum speed training stimulus, sprints of the distance investigated in this study (60 m), should de-emphasize the initial acceleration and be performed with a gradual build-up of speed to reach a peak. For example, the athlete could commence a sprint with a gradual build-up of speed, then once near maximum speed, aim to sprint at 100% intensity for approximately 10-m. The exact distance required to reach top speed from a gradual build-up is likely to vary among individuals. Therefore, coaches and athletes should experiment with the run-in distance to avoid excessively long and fatiguing efforts that could detract from overall session quality. In addition, exposure to maximum speeds can be achieved by performing maximum effort accelerations from a “rolling” start. This will allow athletes to commence sprints in a more upright posture, and replicates the sprinting demands of match play.

When sub-maximum speeds are prescribed for interval training to field-sport athletes, there can be a significant error in achieving the prescribed speed. In the present study, the athletes tended to over-estimate their peak speed and produce more error in the 75% of maximum speed runs, but there was a large variation among individuals. Therefore, during interval training sessions, coaches should provide feedback to athletes on peak speeds reached after each repetition. Measurement of time taken to complete a repetition gives insight into the average speed achieved, but determining the peak speed is more challenging. To do this accurately, instantaneous speed should be recorded during each run with a device such as a global positioning system unit, which can be worn in a pouch situated on the upper back. Such a system has been shown to possess good test and retest reliability with maximum sprints performed by field-sport athletes [26]. To provide feedback after every repetition, live tracking is required.

There are limitations to this present investigation which should be acknowledged. The constraints of the applied setting did not allow for the randomization of conditions, which may have impacted the result. Additionally, the instructions given for the gradual acceleration were not strictly controlled. Nonetheless, these findings are representative of the training environment and therefore hold considerable ecological validity. 

## 5. Conclusions

The results of this study indicated that the gradual build-up to maximum speed produced a greater peak speed than the maximum acceleration run, with most participants demonstrating this trend. For the sub-maximum runs, the average percentage of maximum speed reached was 78 ± 6% for the 75% prescribed run and 89 ± 5% for the 90% prescribed run, with large errors produced by some individuals. It is concluded that sprint training for maximum speed should be performed with a gradual build-up of speed rather than a maximum acceleration, and for sub-maximum interval training, feedback on peak speeds reached should be provided where possible to assist athletes in reaching prescribed speeds.
